# Effectiveness of Multistrain Probiotic Formulation on Common Infectious Disease Symptoms and Gut Microbiota Modulation in Flu-Vaccinated Healthy Elderly Subjects

**DOI:** 10.1155/2022/3860896

**Published:** 2022-01-27

**Authors:** Anna Sandionigi, Alessandra De Giani, Francesco Tursi, Angela Michelotti, Enza Cestone, Silvana Giardina, Jessica Zampolli, Patrizia Di Gennaro

**Affiliations:** ^1^Department of Biotechnology and Biosciences, University of Milano-Bicocca, Milano, Italy; ^2^Complife Italia Srl, San Martino Siccomario (PV), Italy

## Abstract

The decline of the immune system with aging leads elderly people to be more susceptible to infections, posing high risk for their health. Vaccination is thus important to cope with this risk, even though not always effective. As a strategy to improve protection, adjuvants are used in concomitance with vaccines, however, occasionally producing important side effects. The use of probiotics has been proposed as an alternative to adjuvants due to their efficacy in reducing the risk of common infections through the interactions with the immune system and the gut microbiota. A placebo-controlled, randomized, double-blind, clinical trial was carried out on fifty elderly subjects, vaccinated for influenza, to determine the efficacy of a probiotic mixture in reducing common infection symptoms. The incidence of symptoms was evaluated after 28 days of probiotic intake (namely, T28) and after further 28 days of follow-up (namely, T56). The number of subjects, as well as the number of days with symptoms, was remarkably reduced at T28, and even more at T56 in the probiotic group. Furthermore, the influence of probiotics on immunological parameters was investigated, showing a significant positive improvement of total antioxidant capacity and *β*-defensin2 levels. Finally, faecal samples collected from participants were used to assess variations in the gut microbiota composition during the study, showing that probiotic intake enhanced the presence of genera related to a healthy status. Therefore, the collected results suggested that the treatment with the selected probiotic mixture could help in reducing common infectious disease symptom incidence through the stimulation of the immune system, improving vaccine efficacy, and modulating the composition of the resident gut microbiota by enhancing beneficial genera.

## 1. Introduction

Immunosenescence, i.e., the age-related immune system decline, is associated with increased susceptibility to bacterial and viral infections [1]. Respiratory and gastrointestinal tract illnesses, typical diseases associated with the winter season, are a predominant cause of morbidity and mortality in the elderly, as a consequence of the decline of their innate and adaptive immune response [2, 3]. This has considerable implications also considering that emerging infectious diseases, such as COVID-19, seem to have a disproportionately larger effect on older subjects [4]. In this portion of population, the vulnerability is mainly linked to a poor systemic reaction caused by the dysregulation of several factors such as the reduced cellular immune response (due to a decreased function of natural killer cells) [5], alterations in the number of circulating monocytes and dendritic cells [6, 7], reduced phagocytic activities of neutrophils [8], and impaired antibody production [9]. The predisposition to infections is also related to a larger systemic inflammatory state associated with aging, often referred to as “inflammaging,” defined as the chronic low-grade inflammation typical of aging [10] characterized by the continuous production of inflammatory cytokines [11].

Due to all the above-mentioned reasons, even taking part in a vaccination scheme does not completely exempt aged people to contract influenza [12]. Since the efficacy of influenza vaccines commonly reaches only 30–50% in this population, to achieve a wider protection, different strategies have been implemented such as higher antigen dose and intradermal administration [13]. In order to specifically target the aged immune system, recent efforts have been focusing on an alternative approach to increase the efficacy of influenza vaccination that involves administration of adjuvants, such as aluminium salts, squalene, or monophosphoryl lipids, at the time of vaccine inoculation [14]. However, the majority of these compounds present side effects that make their use difficult in such frail subjects, since they promote local inflammation (to boost the immune system) in subjects where chronic inflammation is already ongoing, thus leading to poor vaccine response [15]. For this reason, the development of newer and more specific adjuvants is needed.

Among the changes affecting the elderly, also, the composition of the gut microbiota, which contributes to the local and systemic immune defences, undergoes age-dependent modifications and therefore could have an impact on the incidence and severity of the symptoms of common infections like influenza [16, 17]. Therefore, driving the gut microbiota composition of elderly people toward a healthier one could represent an interesting approach, ameliorating the protective effects of influenza vaccination in aged people. It is well recognized that diet, host genetics, lifestyle, and other external factors can modulate the composition and the metabolic activity of the human gut microbiota, which in turn can impact health [18–20]. The use of prebiotics, probiotics, and synbiotics is becoming a widespread approach to achieve modulation of the gut microbiota by a direct interaction with the host immune system or indirectly by reequilibrating the gut microbiota [21, 22]. In particular, it has been suggested that probiotic administration has a protective effect against infectious diseases by several mechanisms such as secretion of antimicrobial substances, competitive exclusion of pathogens, maintenance of mucosal integrity, and stimulation of systemic or mucosal immune responses [23–25]. Probiotic species have shown a positive effect on duration or frequency of respiratory and gastrointestinal infections, and in reducing the risk of common infections, including respiratory, diarrheal, and musculoskeletal conditions in elderly people [26–29]. Their effect was also evident in increasing the immune response to influenza vaccination in the elderly, suggesting the possible use of probiotic strains as adjuvant that could improve the vaccine efficacy [30–32]. The majority of these evidences are reported for species of *Lactobacillus*, in spite of the fact that one of the main characteristics of microbiota in the old age is the decrease of *Bifidobacterium* species and that higher bifidobacterial levels are correlated with health and longevity [33–35]. Therefore, there is still a need for more scientific evidence, especially from clinical trials, to evaluate the properties of bifidobacteria regarding the influence on intestinal microbiota of older people.

In this randomized, double-blind, placebo-controlled study, we investigated the effect of a probiotic mixture, with the prevalence of bifidobacteria strains, in reducing common infectious disease (CID) episodes during the winter season, in influenza-vaccinated free living elderly subjects. CIDs were monitored either during the product administration or in a follow-up period. The activity of the immune system was monitored in two different districts: at the gastrointestinal level, by measuring faecal calprotectin and faecal *β*-defensin2, and in the respiratory tract, through salivary IgA and salivary total antioxidant capacity (TAC) evaluation. Furthermore, the effect of the probiotic mixture in modulating the gut microbiota was evaluated on faecal samples collected from participants.

## 2. Materials and Methods

### 2.1. Ethics, Approval, and Consent

This study was performed in compliance with the Helsinki Declaration (1964) and its amendment, and the Guidelines on Good Clinical Practice adopted by the International Conference on Harmonisation of Technical Requirements for Registration of Pharmaceuticals for Human Use. The study protocol was reviewed and approved by the “Independent Ethical Committee for Non-Pharmacological Clinical studies” (Genova, Italy) Ref. 2018/14. All subjects provided written informed consent before starting the study.

### 2.2. Study Subjects

Fifty healthy free-living subjects of both sexes, aged between 60 and 80 years, inoculated with influenza vaccine, were enrolled according to a list of inclusion and noninclusion criteria reported as follows. Inclusion criteria were as follows: able to comply with all the trial procedures; inoculated with an influenza vaccine; with body mass index (BMI) 18.5-24.99; willingness to not vary the normal daily routine (i.e., lifestyle, physical activity, etc.); willingness to not alter their usual diet or fluid intake during the trial periods; subjects who have not been recently involved in any other similar study; willingness to follow the proposed alimentary supplement for all the study time; willingness to use during all the study period only the product to be tested; willingness to not use products likely to interfere with the product to be tested; subject aware of the study procedures and having signed an informed consent form. Not inclusion criteria were as follows: contraindications to influenza vaccinations; undergoing treatment related to immune system modulation in the past four weeks; treatment with therapy for immunosuppressant that is being lived more than two weeks or has only been stopped less than three months before the study; received influenza vaccination less than one year before; under a current antibiotic administration; known history of chronic medical condition such as congenital heart disease, liver or kidney disease, or immune deficiency; treatment with probiotics in the six months preceding enrollment; severe concurrent diseases; drug abuse; alcohol abuse; use of fiber products within last six weeks; dietary intake exceptionally high in plant-based, high-fiber foods, including those following a strict vegetarian diet (high-fiber foods: fruits, vegetables, beans, whole grains, and fortified foods).

### 2.3. Study Design

A randomized, double-blind, placebo-controlled, parallel group study was carried out at Complife Italia Srl facilities during the winter season of 2019. Eligible volunteers were equally assigned to the probiotics or the placebo group (25 subjects in the active group and 25 in the placebo group), according to a previously prepared randomization list generated by the study director by using an appropriate statistical algorithm (“Wey's urn”). The study lasted a total of 56 days, including 28 days of treatment (product intake), and further 28 days as a follow-up period. Accordingly, clinical visits were performed at the beginning of the study (T0) at the end of the treatment period (T28), and after the follow-up (T56). During visits, faecal and saliva samples were collected; testing products and daily diaries were distributed at T0.

### 2.4. Study Products

Study products consisted of food supplements in forms of sticks. Probiotic formulation contained 1 × 10^9^ CFU of *Lactiplantibacillus plantarum* subsp. *plantarum* (formerly *Lactobacillus plantarum*) PBS067, 1 × 10^9^ CFU of *Bifidobacterium animalis* subsp. *lactis* BL050, 1 × 10^9^ CFU of *Bifidobacterium longum* subsp. *infantis* BI221, 1 × 10^9^ CFU of *Bifidobacterium longum* subsp. *longum* BLG240, and common excipients used in food supplements. Placebo formulation contained only excipients. All the strains contained in the probiotic formulation were isolated from samples of human origin, and they were provided by Roelmi HPC (Origgio, Italy). Subjects were instructed to take one stick per day of food supplement or placebo, away from meals, in a glass of nonsparkling water, for 28 days. Such a supplementation period was chosen to ensure optimal activity of the supplement used in the study, since approximately 15 days are needed for consistent gut colonization by the probiotic strains selected, i.e., stable detection in faecal content [36].

### 2.5. Study Clinical Parameters

During the study, clinical and immunological parameters were monitored to determine the effectiveness of the probiotic consumption in the reduction of infections and the stimulation of the immune system. As a clinical parameter, the incidence of common infectious disease episodes (CID) was assessed. Immunological parameters examined were concentration of faecal human *β*-defensin2 (HBD-2) and faecal calprotectin of salivary immunoglobulin A (IgA) and salivary total antioxidant capacity (TAC). Furthermore, the overall profile of the gut microbiota was studied to evaluate the modulation effect of the food supplement.

#### 2.5.1. Common Infectious Disease (CID) Episode Symptoms

Enrolled subjects were individually and extensively instructed by the physician on how to report CID on the diary, with a comprehensive description of the symptoms and the duration of the disease episodes. CID symptoms were differentiated in respiratory symptoms (RI) (i.e., cold, cough, sneezing, sore/itchy throat, nasal obstruction, and with or without fever), gastrointestinal symptoms (GI) (i.e., vomiting, diarrhea, and abdominal pain), and musculoskeletal symptoms (MS) (i.e., tension headaches, pain, weakness, stiffness, joint noises, and decreased range of motion). A day with concomitant CID symptoms was computed as one. The number of days and the number of subjects with at least one CID were calculated.

#### 2.5.2. Immunological Parameters

Saliva samples were collected from subjects at the medical centres at T0, T28, and T56. Samples were centrifuged at 3000 rpm at 4°C, for 15 min; supernatants were immediately aliquoted and stored at −20°C. Salivary IgA concentration was measured in all participants by a commercially available ELISA kit (Dia.Metra, Milano, Italy), according to the manufacturer's protocol. Salivary total antioxidant capacity (TAC) was assessed by the Ferric Reducing Antioxidant Parameter (FRAP) assay [37].

Faecal samples were collected by the volunteers at their home at T0, T28, and T56 and kept in the fridge until delivery to the medical centre, where faecal mass was homogenized by vortex mixing and aliquots were stored at –20°C or –80°C. Human *β*-defensine2 (HBD-2) levels were measured with an ELISA kit according to the manufacturer's instructions (Immundiagnostik, Bensheim, Germany). Faecal calprotectin levels were measured using the PhiCal ELISA Test (NovaTec Immunodiagnostica, Dietzenbach, GmBH, Germany) according to the manufacturer's instructions.

### 2.6. Gut Microbiota Analysis

Faecal sample aliquots (1 g) were maintained into Stool Nucleic Acid Collection and Preservation Tubes (Norgen Biotek Corp.) until DNA extraction, performed using the Stool Nucleic Acid Isolation Kit (Norgen Biotek Corp.) according to the manufacturer's instructions. Faecal nucleic acid concentration was evaluated through fluorometric analysis using the Qubi 4 Fluorometer and Qubit™ 1X dsDNA HS Assay Kit (ThermoFisher Scientific, USA). Samples diluted to reach a final concentration of 5 ng/*μ*L were then sent to the sequencing service (Biodiversa S.r.l.). The V3-V4 region of the 16S rRNA gene was used for microbiota analysis. Gene amplification was achieved using PCR primers 341F (5′-CCTACGGGNGGCWGCAG-3′) and 805R (5′-GACTACHVGGGTATCTAATCC-3′) with Illumina library adaptors. Microbial samples were sequenced with Illumina MiSeq 2 × 300 paired-end chemistry (MiSeq Reagent Kit v3). The raw paired-end FASTQ reads were imported into the Quantitative Insights Into Microbial Ecology 2 program (QIIME2, ver. 2020.2.01) [38] and demultiplexed using the native plugin. The Divisive Amplicon Denoising Algorithm 2 (DADA2) [39] was used to quality filter, trim, denoise, and mergepair the data and remove chimeric sequences. The resulting Amplicon Sequence Variants (ASVs) with less than a 50x coverage were discarded from further analyses. The classification of the obtained ASVs was run using the feature-classifier plugin [40], implemented in QIIME2 against the SILVA SSU nonredundant database (138 release) [41], adopting a consensus confidence threshold of 0.8. The analysis on the bacterial diversity, and the corresponding figures, was done using the *phyloseq* R package [42]. Microbiota diversity was described in terms of within (*α*) and between (*β*) sample diversities. The Shannon index and observed features *α*-diversity metrics were calculated to estimate the variation of bacterial diversity at the different time points for the probiotic group and the placebo group. Values were compared using the pairwise Kruskal-Wallis test. *β*-Diversity was calculated using the *vegan* R package [43]. We estimated the weighted and unweighted UniFrac dissimilarity indexes, by sampling 10,000 reads per sample [44]. Statistical significance among groups, including sampling site and developmental stage, was determined by a permutation-based ANOVA (PerMANOVA) test using ADONIS [45] and a 999 permutation-based *β*-diversity distance metrics. PerMANOVA pairwise contrast was performed by the beta-group-significance command of adonis-pairwise function. The structure of microbial communities was explored by nonmultidimensional scaling (NMDS), an ordination approach [46]. The variation in terms of abundance of each ASV was estimated using the DeSeq2 R package [47] performed between the two groups considered during the study and considering the comparison for each pair of times measured.

### 2.7. Statistical Analysis

The difference of subjects with at least one CID between groups was evaluated using *χ*^2^, while the Fisher exact test with False Discovery Rate adjustment for multiple comparisons was used for the analysis by study periods. The number of days with CIDs was compared between groups using the Wilcoxon test. False Discovery Rate adjustment for multiple comparisons was executed for the analysis by study periods. To assess whether the immunological parameters were affected by the treatment during the three different time points, a LMER (Linear Mixed Effect Model) test was performed for each parameter (*β*-defensin2, IgA, and TAC) using the function implemented in the lme4 R package [48]. Following this primary analysis (if significant), some post hoc analyses were performed. During comparisons, the *p* value adjustment was performed with the Tukey method. Immunological parameters without a normal distribution were log-transformed, and the corresponding values were used as response variables in the model. All the models used for the statistical analysis are reported in an explicit form in the Supplementary Materials.

## 3. Results

### 3.1. Subjects of the Study

Fifty influenza-vaccinated healthy free-living elderly subjects were randomly and equally assigned to the probiotic group (*N* = 25) or to the placebo group (*N* = 25). All subjects completed the study, and products were well tolerated.  Table 1 reports baseline characteristics of the total population. Results showed the homogeneity of parameters in the two groups in terms of age and body mass index.

### 3.2. Clinical Outcome

The incidence of common infectious disease episodes (CID) was assessed as the number of subjects that experienced at least one CID episode, and as the number of days of episodes, all over the study period, and the two different stages of the study: the treatment period (T0-T28) and the follow-up period (T28-T56). Accordingly, Table 2 shows the number of subjects who experienced at least one CID symptom in the two groups, as well as the specific CID symptom category, defined as respiratory (RI), musculoskeletal (MS), and gastrointestinal (GI) symptoms. Considering the entire study period, the probiotic administration resulted in a significant decrease (OR = 0.22, with 95%CI = 0.06‐0.73; *p* value < 0.05) of the number of subjects presenting CID with respect to the placebo: 10 vs. 19, corresponding to 40% vs. 76% of the population, respectively. Analysing the outcome of the study, a reduction during the treatment period was recorded in the probiotic group (8 subjects vs. 13 in the placebo). The decrease became significant (*p* value < 0.05) in the follow-up period, where only 5 volunteers in the probiotic group presented symptoms with respect to 14 in the placebo. As some subjects experienced CID symptoms in both periods, the total number of subjects with CID does not correspond with the mathematical sum obtained for each study period (treatment+follow-up). The proportion of subjects with CID in the category of three symptoms throughout the two periods also indicated an overall reduction of cases in the probiotic group, pointing out a prolonged effect achieved by the treatment.

A similar trend was observed by comparing the distribution of the number of days with CID symptoms between the two groups (Table 3). Subjects in the placebo group experienced CID symptoms for a total of 224 days (32%), whereas the presence of symptoms was considerably and statistically lower in the probiotic group (a total of 103 days, 14.7%; *p* value < 0.01), suggesting an overall effect of the probiotic treatment throughout the study. Furthermore, a significant reduction (*p* value < 0.01) of the number of days with CIDs was achieved in the follow-up period (38 vs. 124 days), once more pointing out a long-lasting effect of the probiotic treatment. As before, the number of days during which subjects felt more than one category of symptoms was computed as one day.

### 3.3. Immunological Parameters

The effect of the treatment on the immune system was evaluated taking into account several immunological parameters linked to the respiratory and the gastrointestinal tract, i.e., levels of salivary immunoglobulin A (IgA) and salivary total antioxidant capacity (TAC): faecal *β*-defensin2 (HBD-2) and faecal calprotectin, respectively. The outcomes related to the levels of the immunological parameters are reported as a boxplot, representing the total distribution in the subjects within the specific group of treatment.

**Figure 1:**
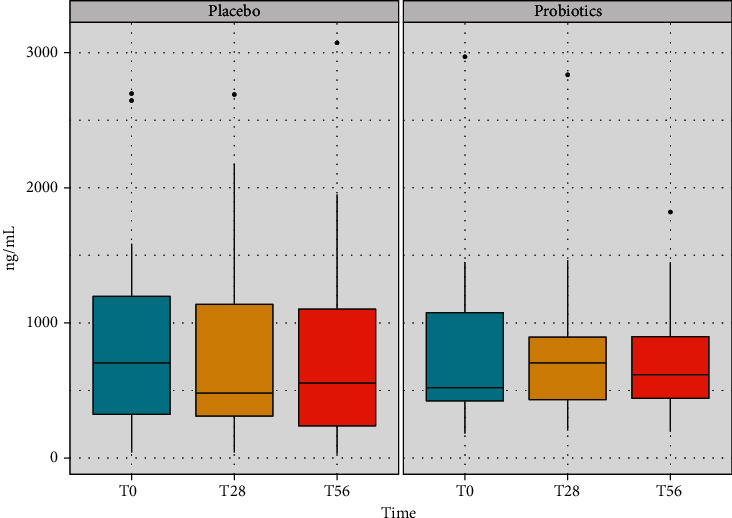
Concentration of IgA recorded in saliva samples of volunteers in the placebo and in the probiotic groups at the three time points of the study (baseline, after the treatment period, and after the follow-up). The boxplots show the minimum, the first quartile, the median, the third quartile, and the maximum of the IgA values collected.

Levels of salivary IgA measured at the end of the placebo intake (T28) and after the follow-up period (T56) did not show any significant variation with respect to the basal level, (Figure 1). A similar profile is evidenced also in the probiotic group, considering the same time points. However, taking into account the variation of the median value, it is possible to point out an increasing trend in the levels of the salivary IgA, above all at the end of the administration period. Indeed, at T28, the levels of the immunological parameter were around 27% higher compared to the baseline. The positive trend was maintained after the follow-up period, where an increment of the 21% respect to T0 was recorded (Figure 1). Nevertheless, no significant differences were observed between the placebo and probiotic groups at any time point.

**Figure 2:**
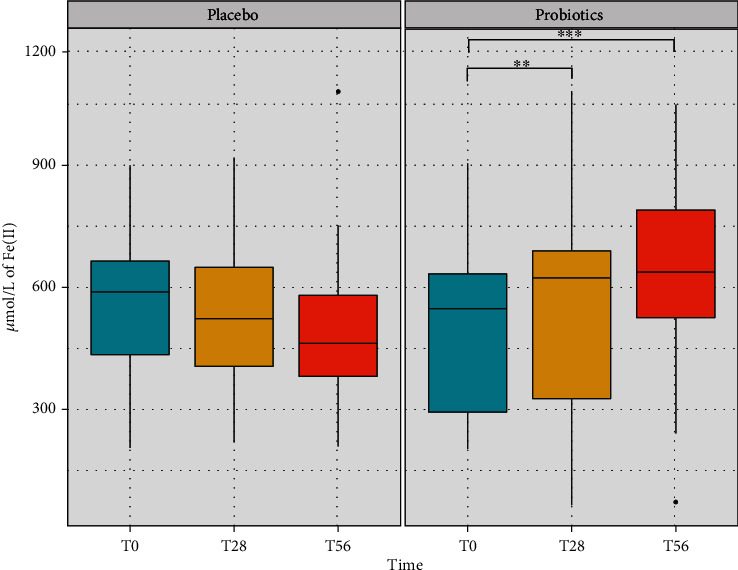
Level of total antioxidant capacity (TAC) recorded in the saliva samples of volunteers in the placebo and in the probiotic groups at the three time points of the study (baseline, treatment, and follow-up). The activity was determined by the Ferric Reducing Ability of Plasma (FRAP) method and expressed as *μ*mol/L of Fe (II) reduced. The boxplots show the minimum, the first quartile, the median, the third quartile, and the maximum of the FRAP values collected. Statistical differences were calculated using the Linear Mixed Effect Model (LMER): ^∗∗^*p* value < 0.01 and ^∗∗∗^*p* value < 0.001.

Total antioxidant capacity was determined by the Ferric Reducing Ability of Plasma (FRAP) method. The activity was expressed as *μ*mol/L of Fe (II) reduced. Placebo intake did not show any significant variation of the parameter compared to the basal level at both T28 and T56 (Figure 2). On the contrary, probiotic mixture intake increased significantly FRAP levels after the treatment period (*p* value < 0.01) compared to baseline values, and even more after the follow-up (*p* value < 0.001) (Figure 2). The increment in FRAP levels during time is attributable to the active product (i.e., probiotics) intake, even though no significant difference was determined comparing the group with the placebo.

**Figure 3:**
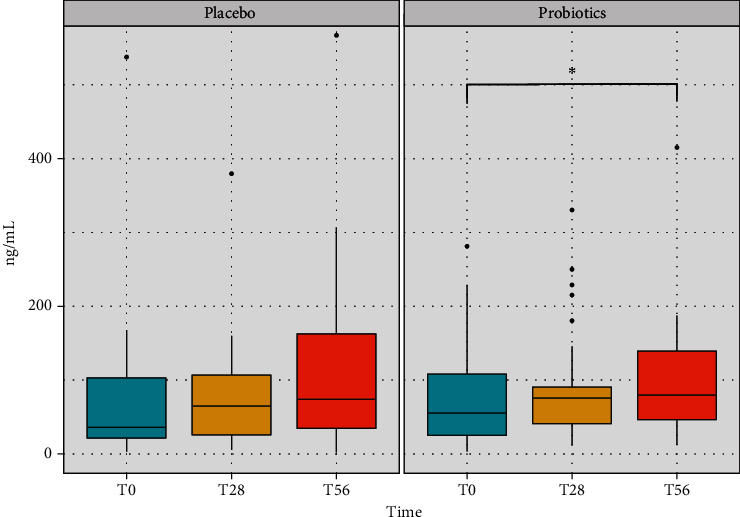
Concentration of *β*-defensin2 levels recorded in the faecal samples of the volunteers in the placebo and in the probiotic groups at the three time points of the study (baseline, treatment, and follow-up). The boxplots show the minimum, the first quartile, the median, the third quartile, and the maximum of the *β*-defensin2 values collected. Statistical differences were calculated using the Linear Mixed Effect Model (LMER): ^∗^*p* value < 0.05.

Regarding the levels of *β*-defensin2, no significant differences were appreciated in the placebo group throughout the study (Figure 3). Instead, the probiotic group showed a progressive increase of the *β*-defensin2 level either at the end of the treatment or after the follow-up period, achieving at T56 a significant difference (*p* value < 0.05) with respect to the beginning of treatment (Figure 3). From the results, it is possible to consider a potential effect of the probiotics in the stimulation of the release of the molecules by the intestinal cells.

Concerning the other faecal parameter, i.e., calprotectin, there were no significant effects (data not shown) due to the intervention, and the levels in the probiotic group and in the placebo group were quite similar. Interestingly, the levels were well below the limit of 45 *μ*g/g stool, which is considered as a normal value in subjects older than 65 years [49].

### 3.4. Gut Microbiota Analysis

**Figure 4:**
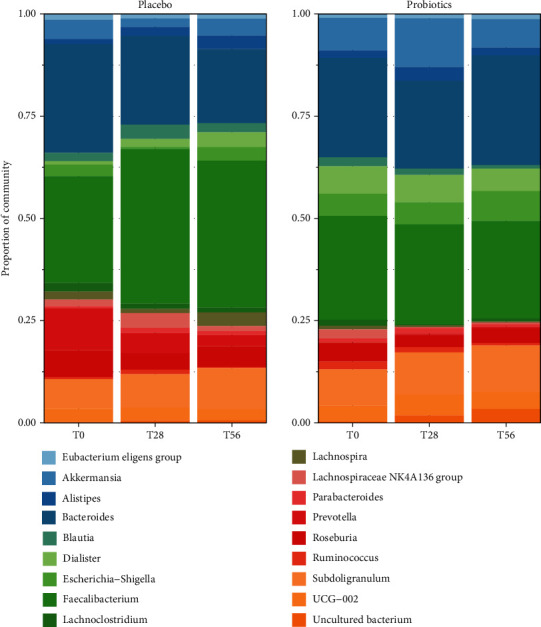
Taxa bar plot representing the most representative genera composing the faecal microbiota in elderly subjects identified by V3-V4 16S rRNA amplicon sequencing during the study period (T0-T28-T56). The cumulative relative abundance of specific ASVs belonging to the probiotics or to the placebo groups is shown: each bar represents a study period and each colored box a bacterial taxon. The order from the top to the bottom of each ASVs at the phylum level corresponding to a different colored box represents the percentage of relative abundance of bacteria within the group.

After gut microbiota sequencing and DADA2 filtering, a total of 10.800.549 number of reads was obtained (65.457 average reads per sample) corresponding to 11.465 ASVs (Amplicon Sequence Variants). The taxonomic assignment resulted in a total of 12 phyla, 21 classes, 54 orders, 107 families, and 248 genera. The dominant phyla present in the samples were Firmicutes and Bacteroidota, followed by Proteobacteria, Verrucomicrobia, and Actinobacteria. Considering the relative abundances across the different time points, the most represented genera were *Bacteroides*, *Faecalibacterium*, *Prevotella*, *Subdoligranulum*, *Dialister*, and *Akkermansia*, which are highly present in both groups before, during, and after the treatment (Figure 4).

**Figure 5:**
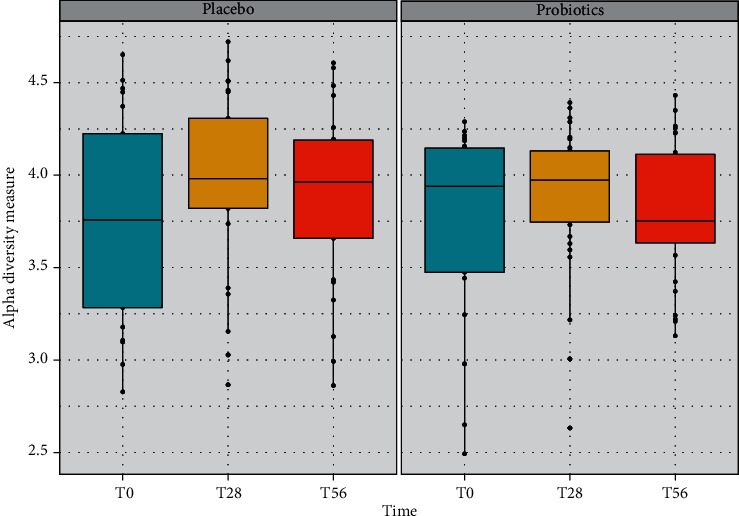
Bacterial *α*-diversity analysed in the faecal samples of the volunteers in the placebo and in the probiotic groups at the three time points of the study (baseline, treatment, and follow-up). Analyses are based on the Shannon index using a multiple group test (Kruskal-Wallis test at two levels of significance).

Bacterial *alpha*-diversity was analysed to estimate the changes of species richness (biodiversity) within each group at the three time points, considering the number of ASVs present in each sample (Figure 5). In general, the diversity, measured with the Shannon index using a multiple group test (Kruskal-Wallis test at two levels of significance), showed that no significant differences emerged among the two groups in terms of species richness. Accordingly, the Shannon diversity index was also investigated by pairwise difference tests carried out between groups (Wilcoxon signed-rank test) and intragroups (Mann-Whitney test) for the three time points. The pairwise difference tests showed no significant differences between groups at the different periods of the study.

To estimate the changes of biodiversity in terms of species abundances within each treatment group at the three time points, also bacterial *beta*-diversity was investigated. This analysis measures the degree of bacterial biodiversity between the different samples considering the number of species that are not in common in different samples. For this purpose, a multidimensional scaling (MDS) test was carried out. Weighted UniFrac distance was used to estimate community dissimilarity by taking into account the presence or the absence of species in each sample. However, no differences were recorded among the treatment and the placebo during time (Figure S1).

**Figure 6:**
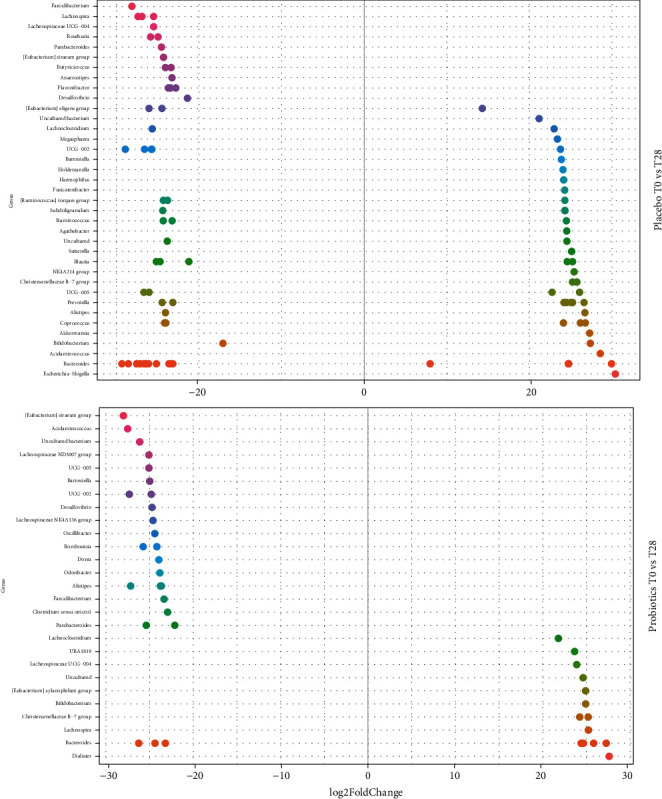
Changes in relative abundances computed through DeSeq2 differential abundance analysis expressed as Log2FC comparison of the placebo and the probiotic group after the treatment (T28). Negative Log2FC represents genera enhanced in the placebo and in the probiotic group, while positive Log2FC represents genera enhanced only in one group. Each point represents an individual ASV assigned at the genus level. To enhance clarity, only those ASVs with *p*‐adj < 0.0001 are shown.

To evaluate the effect of the probiotic formulation intake on the modulation of the elderly gut microbiota, the *ad hoc* analysis DESeq2 was developed to understand how specific taxa varied between the different times in the two groups. Accordingly, the analysis regarding the probiotics and the placebo group during the entire study period was carried out at genus level. Indeed, a certain differentiation between the beginning and the end of the treatment period (T0-T28) was observed in both groups: the genera most relevant for their change during time are reported in Figure 6. Furthermore, changes in the relative abundances observed at the end of the treatment period were still ongoing after the follow-up (Figure S2).

At T28 in the placebo, 37 genera (grouping different ASVs) were reported to significantly vary (adj-*p* < 0.0001) (Figure 6). Interestingly, only 9 genera had a one-way modulation trend, while all the others presented both positive and negative modulations, suggesting that these oscillations were linked to physiological fluctuations. Among the genera that showed only a positive variation, there were, for example, *Escherichia-Shigella, Acidaminococcus*, *Akkermansia*, and a genus related to the family of Chistensenellaceae, while in the group of genera characterized by a negative variation (adj-*p* < 0.0001) were recorded important genera such as *Faecalibacterium*, *Lachnospira*, *Roseburia*, and *Butyricicoccus.* At the same time point, the gut microbiota modulation mediated by the probiotic treatment comprehends 27 genera (grouping different ASVs) that significantly varied (adj-*p* < 0.0001) accordingly to specific trends. The only exception was the genus *Bacteroides*, which showed ASVs varying both in a positive manner and in a negative manner (Figure 6). Specifically, the genera positively affected by the probiotic intake were *Dialister*, *Lachnospira*, *Bifidobacterium*, and *Lachnoclostridium*, while among the taxa negatively influenced there were, for example, *Alistipes*, *Dorea*, *Parabacteroides*, and *Clostridium*. At the end of the follow-up period, the variation trends were similar to the ones of T28 (significantly varied genera (adj-*p* < 0.0001): 39 vs. 22 in placebo and probiotic groups, respectively). Indeed, in the placebo group, there were both positive and negative modulations, while in the probiotic group, the ASV behaviour was clear (Figure S2). A positive variation in the latter group was observed for other interesting genera such as *Butyricicoccus* and *Akkermansia*, while at the same stage in the placebo, there was a negative effect on important genera such as *Bifidobacterium*, *Blautia*, *Lactobacillus*, and *Faecalibacterium* (Figure S2).

## 4. Discussion

Aging is associated with a rise in the incidence of inflammation and the susceptibility to gastrointestinal and respiratory infections [50]. The principal factors causing such condition are immunosenescence and inflammaging, which concur to the dysregulation of the immune system, producing a reduction in the efficacy of vaccination [51]. This has important implications considering not only the increase of aging in the global population but also the impact on the elderly of new infection onset, like the recent SARS-CoV-2 pandemic [52]. In this context, it is clear the necessity of new strategies to counteract the immune system frailty, linked to the old age, and to enhance the vaccination efficacy. An important role in the age-related changes, responsible for the increase of vulnerability, is played by the gut microbiota, which has been identified as a fundamental immune regulator [53]. Several recent studies revealed a correlation between its composition and the vaccination response: its strong ability to modulate host immunity and inflammation could exert a significant effect on vaccination [54]. However, since gut microbiota is compromised in older adults, the use of probiotics has been reported as a valid approach to improve vaccine immunogenicity by modulating the resident microorganisms [55].

In the present study, the effect of a food supplement, containing selected probiotics, on the incidence of common infectious diseases in a flu-vaccinated elderly population was investigated, evaluating also the stimulation of the response of the immune system associated with the respiratory and the gastrointestinal tracts. Furthermore, the modulation of the gut microbiota was considered. Interestingly, a certain influence of the active ingredient on the occurrence of illnesses typical of the winter season was observed. In general, the number of subjects that reported symptoms was significantly lower in the probiotic group, not only during the treatment period but even more throughout the follow-up period. Also, the number of days linked to the presence of CID symptoms in the probiotic group was significantly reduced compared to the placebo group, which instead reported an increase in the duration. This result could suggest a long-lasting effect of the probiotic mixture, even during the follow-up. Indeed, several scientific papers report the efficacy of probiotics in fighting infections, not only in the case of gastrointestinal illnesses, such as those caused by *Clostridium difficile*, but also on the upper respiratory tract condition, mainly caused by group A streptococci [23, 29, 56]. In recent years, it has been reported a reduction in the duration and in the incidence of infections due to probiotic consumption, not only in children and/or healthy adults [57–59] but also in the elderly [60–62]. Results achieved in the present study are in agreement with those results. Nevertheless, the reduction was observed within a shorter period of administration (4 weeks) with respect to those studies (ca. 12 weeks). Such results suggest a protective effect exerted by the probiotic mixture that began during the treatment period and lasted in the follow-up period, whereas placebo treatment left volunteers with the common winter illnesses.

The role of probiotics in the decrease of infection incidence is mainly related to their ability to modulate the immune system response by several mechanisms including innate (like phagocytic or natural killer cell activity), adaptive (immunoglobulins production), and local (improved gut barrier and cytokine production) immune functions [63, 64]. It is also well known that through the same mechanisms, probiotics have been indicated to enhance vaccine effect [65]. In this context, one of the objectives of this study was to evaluate the effect of the active treatment on specific markers related to the systemic inflammation induced by infections. *β*-Defensin2 levels resulted an increase after probiotic intake, achieving significant variation in the follow-up period, if compared to baseline (T0). Indeed, *β*-defensins are antimicrobial peptides playing an important role in protection against pathogens and in the modulation of the inflammation through the reduction of the proinflammatory and the increase of the anti-inflammatory cytokines [66, 67]. Even though these proteins are produced by epithelial cells, their release can be induced and increased by gut microbiota. Indeed, there is evidence that probiotic bacteria can influence innate immunity through *β*-defensin expression [68, 69]. Also, TAC levels resulted significantly increased in the probiotic group both after one month of treatment and during the follow-up compared to baseline. Generally, a systemic functional loss related to aging has been associated also with an increase of reactive oxygen species (ROS). The resulting oxidative stress due to this excess induces cellular senescence and consequently structural damage. Thus, it is considered one of the major causes of aging [70]. It has been reported that antioxidant defence system activity, comprising low molecular weight antioxidants (i.e., glutathione, ascorbic acid, and tocopherols) and antioxidant enzymes, especially the superoxide dismutase (SOD), decreases with age [71]. Among the beneficial effects exerted by probiotics, the influence on antioxidant capacity has been reported. Indeed, due to their oxygen sensitivity, probiotics exhibit a rapid oxidative stress response that can be exerted also in the host by promoting the production of antioxidant enzymes to help remove ROS [72].

In general, it is hard to define a typical gut microbiota composition of elderly, since it is highly variable, depending on several factors such as healthy status, lifestyle, medical treatment (i.e., use of antibiotics), and environmental factors (i.e., living situation) [17]. However, a number of studies reported observations on the changes the microbiota undergoes with aging, such as reduction in biodiversity (that increases with frailty), decrease of beneficial bifidobacteria, and increase of harmful Enterobacteriaceae [73–75]. The results related to the gut microbiota analysis obtained in this study are in line with these findings. In general, dominant phyla found in the faecal samples of both studied groups were all typical of adult gut microbiota [76]. Regarding the bacterial biodiversity, the probiotic intake did not induce any important significant changes in the subjects. However, the overall absence of statistically significant differences, neither in the *α*- and *β*-diversity nor in the taxa abundances in the samples during the treatment, was not surprising. Indeed, it is well known that the intestinal microbiota tends to be quite stable during time in healthy subjects and it seems to have a certain resilience to perturbations, unless drastic interventions occur (i.e., after antibiotic treatment) or durable variation in lifestyle (i.e., long-term diet) [77, 78]. This result is also consistent with what was observed in other scientific studies, performed on pre- and probiotics, in which the administration of such products leads to slight or no changes in the macroscopical microbial composition [79–82]. Nevertheless, in our study, a certain effect of the probiotic treatment was observed on the variation rate of some relevant taxa. Results obtained with an *ad hoc* analysis, performed to determine taxa that showed a variation after the treatment, revealed that probiotic product administration seemed to enhance some genera. The most remarkable result was that the positive variations recorded after the treatment were still evident after the follow-up period, suggesting that probiotics may colonize, persist, and exert a long-term effect [36]. Among the taxa enhanced by probiotic intake after 28 days, it was evident the presence of genera that are reported to be beneficial for the host. *Lachnoclostridium*, *Lachnospira*, and Lachnospiraceae UCG-004 are all members of the Lachnospiraceae family of the Firmicutes phylum, which are very well known to be short-chain fatty acid (SCFA) producers, especially butyrate, being able to boost anti-inflammatory capacity of the host, by suppressing the activation of proinflammatory pathways [83]. They have also been reported to facilitate T-reg differentiation and to stimulate TGF*β* and IL-10 production by immune cells [84]. Even the genus *Bifidobacterium* was enhanced by the treatment, though this was expected due to the formulation of the probiotic product. Also, *Bacteroides* genus seemed to change in the probiotics group; however, its variation was both positive and negative. Interestingly, this genus is the object of a huge debate since it has both commensal positive features as well as opportunistic pathogenic characteristics [85]. In general, Bacteroidetes (that comprehends the genus *Bacteroides*) is one of the most abundant phyla of bacteria in the healthy human gut and they have been hypothesized as mammalian symbionts affecting on the immune system [86–89]. Some species of *Bacteroides* are known for the beneficial conversion of succinate to propionate and for the production of sphingolipids that play a role in maintaining homeostasis and modulating inflammation [90, 91]. As for the genus *Christensenellaceae* R-7 group, the family Christensenellaceae has been recently associated with health [92], and it has also been reported as a typical group found in healthy centenary subjects, thus correlating it to longevity [93]. Therefore, an increase of the abundance of this group could be extremely positive for aged people. The same publication [93] reported that *Dialister* and related genera are present in the gut microbiota along lifespan. This genus was particularly enhanced by the probiotic treatment in this study. Even if its role in the gut ecosystem is still to be clarified, its depletion has been noted in people with pathological diseases, such as autistic individuals, and in subjects with allergic diseases [94, 95]. At the same study point (T28), in the placebo group, the variation of important genera such as *Bacteroides*, *Alistipes*, *Prevotella, Bifidobacterium*, *Blautia*, and *Ruminococcus* was observed; however, their variances were both positive and negative, so it is not possible to establish whether it depended on other factors unrelated to the study. In the probiotic-treated group, positive variations of interesting genera, such as *Christensenellaceae* and *Lachnoclostridium*, were still evident after the follow-up period. Furthermore, two more genera resulted enhanced by the probiotic intake: *Akkermansia* and *Butyricicoccus*. The genus *Akkermansia* is well known for the beneficial effects on humans, linked to its ability to restore and maintain intestinal barrier integrity, and it has been considered as a promising candidate as a next-generation probiotic [96–98]. As well, a member of the genus *Butyricicoccus*, the species *B. pullicaecorum* that colonizes the mucus layer of the human colon, seems to be a good candidate for use as a probiotic, since it exerts anti-inflammatory effects, and it is a great butyric acid producer [99].

Overall, the genera enhanced in the probiotic group are all health-associated bacteria. Many of them are capable of producing short-chain fatty acids (SCFAs) that are important metabolites for the good functionality of the gut barrier. Furthermore, they have been reported to exhibit anti-inflammatory responses after a proinflammatory stimulus.

## 5. Conclusion

In conclusion, the results obtained in the present study suggest an effect of the probiotic treatment that could have an impact on the immunological markers linked to infections, being responsible for reducing the incidence of CID episodes during the treatment period. Indeed, when the systemic inflammatory disorders increased due to seasonal changes, the use of the probiotic formulate helped to reduce their frequency, suggesting an immunomodulatory effect that could enhance vaccine efficacy. Furthermore, the selected probiotic mixture could exert a positive influence on gut microbiota, modulating resident bacteria by enhancing beneficial genera.

## Figures and Tables

**Table 1:** Demographic characteristics of subjects at the beginning of the study. Results of age and BMI (body mass index) are reported as mean value ± standard deviation.

	Placebo			Probiotics		
	Total	Female	Male	Total	Female	Male
No. of subjects	25	19	6	25	17	8
Age (years)	64.92 ± 6.04	63.47 ± 5.10	69.50 ± 6.98	62.56 ± 4.93	63.71 ± 5.28	60.00 ± 3.32
BMI (kg/m^2^)	23.59 ± 1.52	23.55 ± 1.58	23.72 ± 1.40	23.25 ± 1.89	23.15 ± 1.78	24.20 ± 0.87

**Table 2:** Number of subjects who experienced at least one common infectious disease (CID) symptom.

	Placebo			Probiotics		
	Total	0-28 d	28-56 d	Total	0-28 d	28-56 d
No. of subjects with at least one CID	19^a^	13	14^b^	10^a^	8	5^b^
No. of subjects with at least one RI		10	9		7	2
No. of subjects with at least one MS		9	8		3	1
No. of subjects with at least one GI		6	4		2	2

The table represents the number of subjects with at least one common infectious disease (CID) symptom during the whole study (total), the treatment period (0-28 days), and the follow-up period (28-56 days). CID are differentiated into the following categories: respiratory (RI), musculoskeletal (MS), and gastrointestinal (GI) symptoms. The difference of total subjects with at least one CID between groups was evaluated using *χ*^2^, while the Fisher exact test (with False Discovery Rate adjustment for multiple comparisons) was used for the analysis of the single study periods. Statistically significant results are reported in bold (*p* value < 0.05). ^a^Indicates the comparison “total in placebo group” vs. “total in probiotic group”; ^b^indicates the comparison “follow-up in placebo group” vs. “follow-up in the probiotic group”.

**Table 3:** Number of days characterized by at least one common infectious disease (CID) symptom.

	Placebo			Probiotics		
	Total	0-28 d	28-56 d	Total	0-28 d	28-56 d
No. of days with at least one CID	224^a^	100	124^b^	103^a^	64	38^b^
No. of days with at least one RI		87	71		54	20
No. of days with at least one MS		60	52		9	6
No. of days with at least one GI		13	31		8	12

Number of days characterized by at least one common infectious disease (CID) symptoms during the whole study (total), the treatment period (0-28 days), and the follow-up period (28-56 days). CID are differentiated into the following categories: respiratory symptoms (RI), musculoskeletal symptoms (MS), and gastrointestinal (GI) symptoms. The number of days with CIDs was compared between groups using the Wilcoxon test (with False Discovery Rate adjustment for multiple comparisons for the analysis by study periods). Statistically significant results are reported in bold (*p* value < 0.01). ^a^Indicates the comparison “total in the placebo group” vs. “total in the probiotic group”; ^b^indicates the comparison “follow-up in the placebo group” vs. “follow-up in the probiotic group”.

## Data Availability

All data generated or analysed during this study are included in this published article and its supplementary information files. The datasets used and analysed during the current study are available from the corresponding author on reasonable request.

## Abbreviations

CID: Common infectious disease episodes
RI: Respiratory symptoms
MS: Musculoskeletal symptoms
GI: Gastrointestinal symptoms
IgA: Immunoglobulin A
TAC: Total antioxidant capacity
FRAP: Ferric Reducing Antioxidant Parameter
ASV: Amplicon Sequence Variant.

## Supplementary Materials

Supplementary 1 Figure S1: nonmultidimensional scaling (NMDS) test. Weighted UniFrac distance was used to estimate community dissimilarity by taking into account the presence or the absence of species in each sample of the placebo and probiotics group.

Supplementary 2 Figure S2: changes in relative abundances computed through DeSeq2 differential abundance analysis expressed as Log2FC comparison of the placebo and the probiotics group after the treatment (T56). Negative Log2FC represents genera enhanced in the placebo and in the probiotic group, while positive Log2FC represents genera enhanced only in one group. Each point represents an individual ASV assigned at the genus level. To enhance clarity, only those ASVs with *p*‐adj < 0.0001 are shown.
