# Supplementary figures and images for: Effectiveness of Multistrain Probiotic Formulation on Common Infectious Disease Symptoms and Gut Microbiota Modulation in Flu-Vaccinated Healthy Elderly Subjects
Source: Biomed Res Int. 2022 Jan 27;2022:3860896. doi: 10.1155/2022/3860896 (PMC8814717; doi:10.1155/2022/3860896)

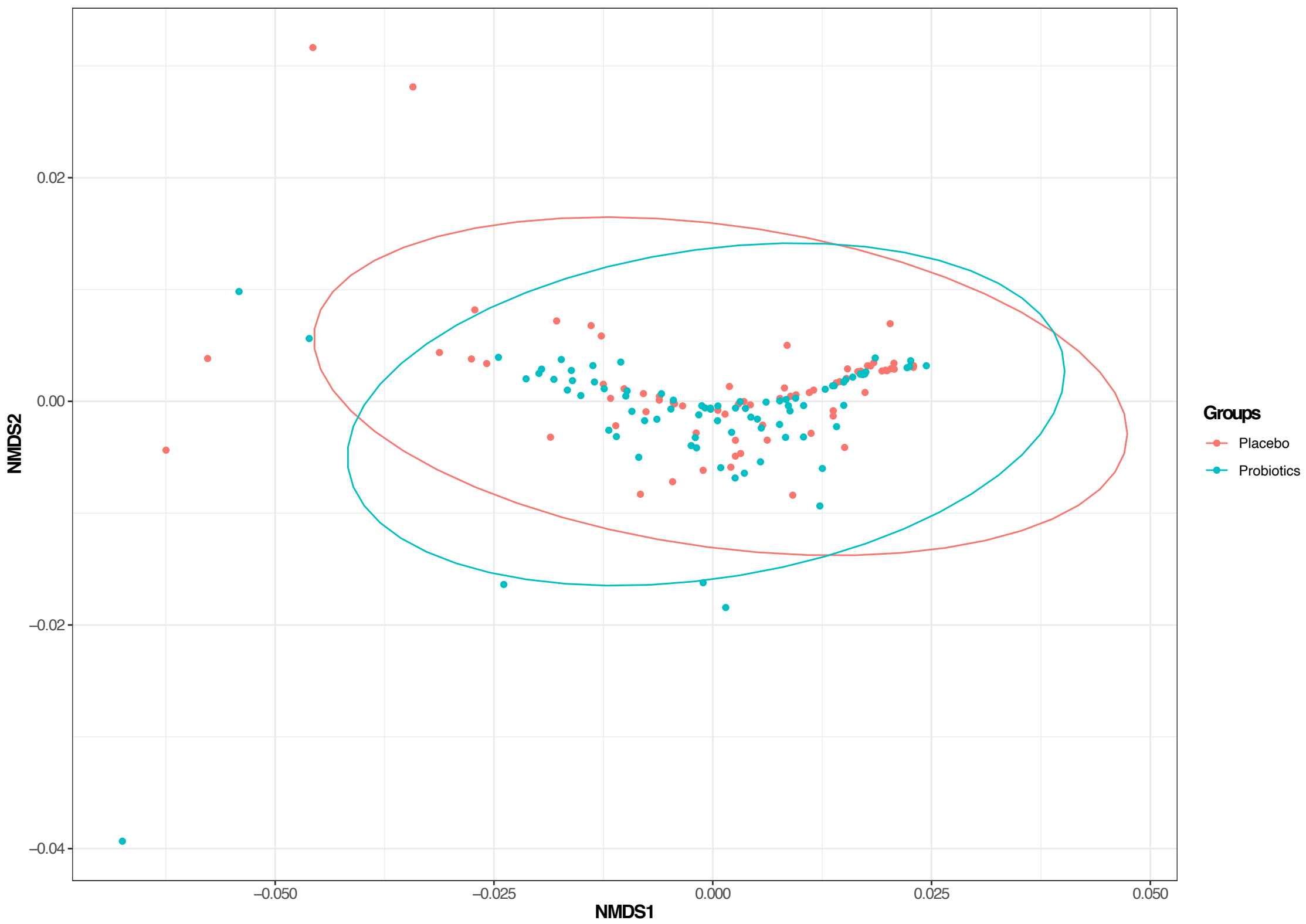

Supplement: Supplementary 1 — Figure S1: nonmultidimensional scaling (NMDS) test. Weighted UniFrac distance was used to estimate community dissimilarity by taking into account the presence or the absence of species in each sample of the placebo and probiotics group. [file 3860896.f1.pdf]

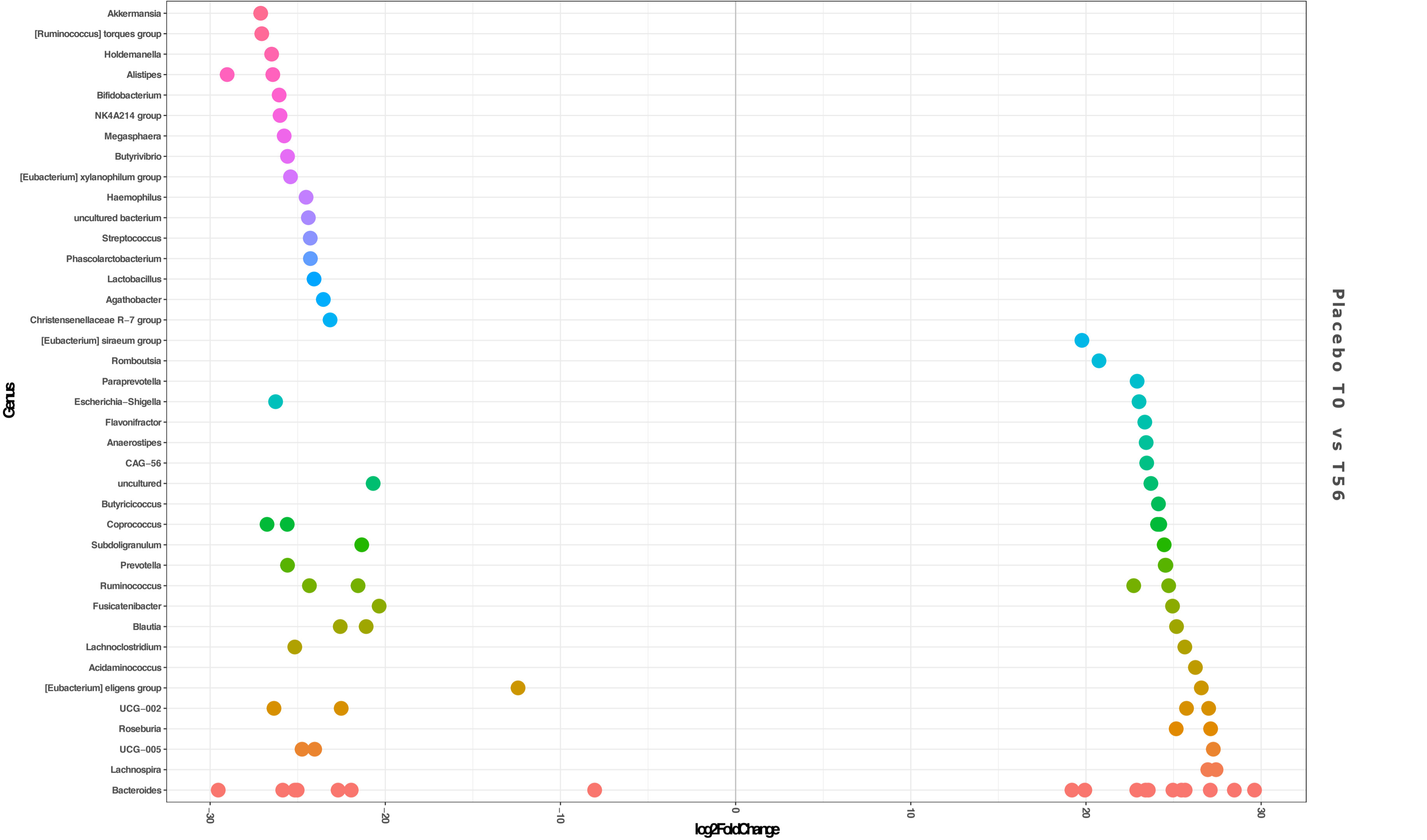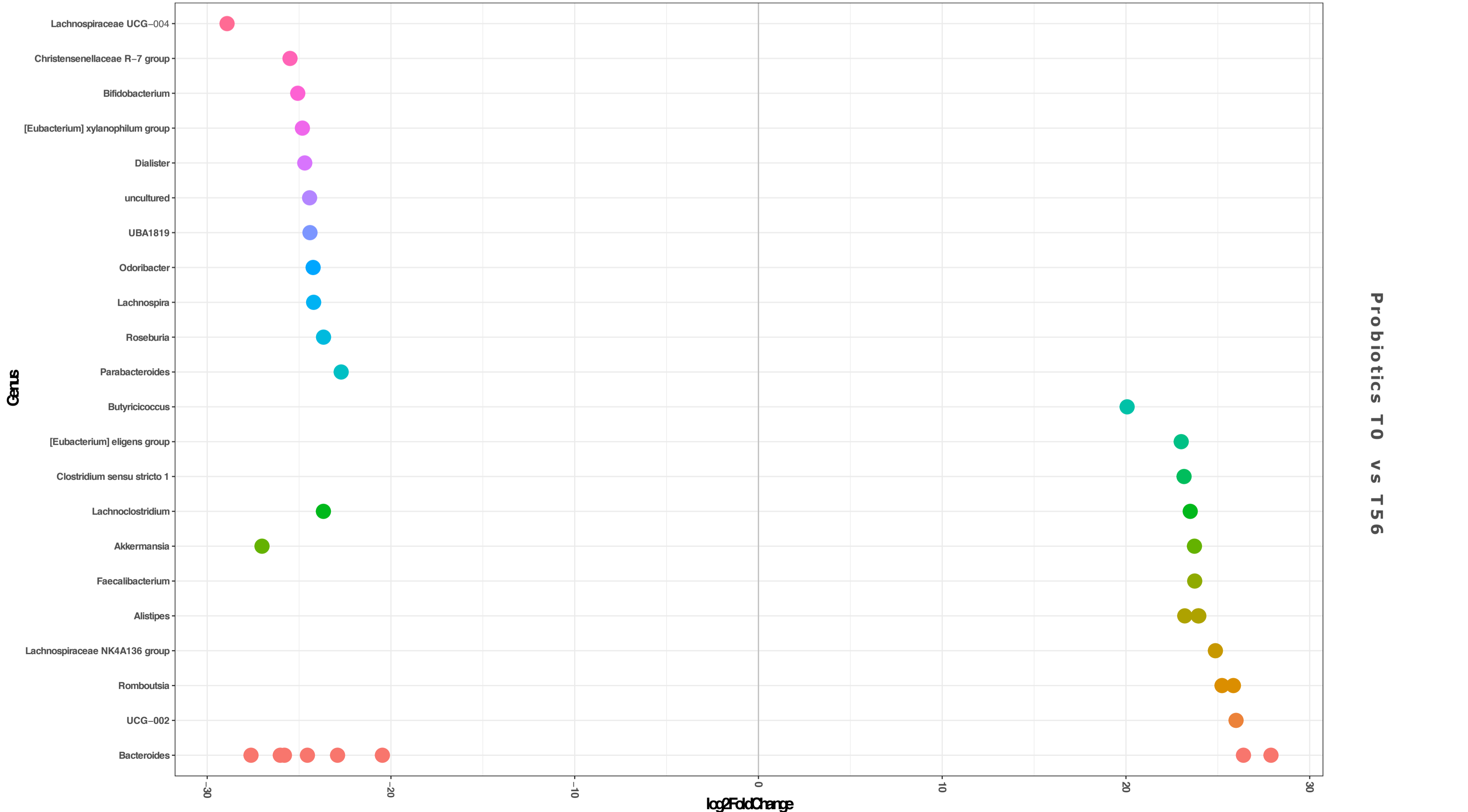

Supplement: Supplementary 2 — Figure S2: changes in relative abundances computed through DeSeq2 differential abundance analysis expressed as Log2FC comparison of the placebo and the probiotics group after the treatment (T56). Negative Log2FC represents genera enhanced in the placebo and in the probiotic group, while positive Log2FC represents genera enhanced only in one group. Each point represents an individual ASV assigned at the genus level. To enhance clarity, only those ASVs with p‐adj < 0.0001 are shown. [file 3860896.f2.pdf]
